# Safety and acute performance of atrial fibrillation ablation using a temperature-controlled, very high-power short-duration catheter and a new radiofrequency generator

**DOI:** 10.1007/s10840-023-01652-3

**Published:** 2023-11-06

**Authors:** Laurent Macle, Girish M. Nair, Allan Skanes, Martin Aguilar, Alfredo Pantano, Yaariv Khaykin, Atul Verma

**Affiliations:** 1 Montreal Heart Institute, Montreal, QC Canada; 2 Université de Montréal, Montreal, QC Canada; 3 University of Ottawa Heart Institute, Ottawa, ON Canada; 4 London Health Sciences Centre, London, ON Canada; 5 Southlake Regional Health Centre, Newmarket, ON Canada; 6 McGill University Health Centre, Montreal, QC Canada

The safety and effectiveness of ablation performed using conventional power temperature-controlled (QMODE; ≤ 50 W) and very high-power short-duration (QMODE+ ; 90 W/4 s) mode with the QDOT MICRO catheter (QDM; Biosense Webster, Inc.) and the nMARQ generator have been reported [1–4]. The nGEN generator was developed to support the full suite of power-controlled, temperature-controlled, and multichannel radiofrequency (RF) ablation catheters. Initial studies for QDM and nGEN were limited to early experience with single or two-center studies, including reports of high rate of catheter tip char with QMODE+ [5, 6]. This prospective study (ClinicalTrials.gov Identifier: NCT04545619) was conducted at 4 Canadian sites to assess feasibility and safety of the QDM catheter with the nGEN generator in the clinical setting.

The study enrolled 40 participants aged ≥ 18 years (mean age 61.7 ± 9.34 years, 62.5% male, mean atrial fibrillation [AF] duration 52.4 ± 51.3 months) with symptomatic paroxysmal or early persistent AF undergoing catheter ablation for pulmonary vein isolation (PVI). Follow-up was performed at 7 days for adverse events and medications and at 3 months for adverse events, medications, medical/hospitalization history, and electrocardiogram. Sites received Institutional Review Board/Ethics Committee approval; all participants signed a patient informed consent form. The primary endpoint was confirmation of entrance block in all targeted pulmonary veins (PVs) after adenosine and/or isoproterenol challenge.

QMODE+ was used primarily for PVI with overlapping 3-mm lesion tags for anterior, inferior, and roof of left atrium and nonoverlapping lesions posteriorly to avoid esophageal heating. QMODE was used at operator discretion for thicker atrial tissue, touch-ups, and at non-PV sites. The first 14 participants underwent treatment with nGEN version 0 (V0); due to catheter tip char noted in other centers, software adjustments were made to the power ramp up to 90 W and the power titration in response to temperature to optimize performance [6]. The updated version, nGEN V1c, was used for the remainder of the study. One participant who did not undergo catheter insertion was excluded from the safety and effectiveness analysis sets, and 1 participant who had only non-PV targets ablated was additionally excluded from the effectiveness analysis set. PV entrance block was confirmed in 97.4% (37/38) of participants in the effectiveness analysis set and in all 37 participants treated with the study catheter alone (nonstudy catheter used in 1 procedure after connectivity troubleshooting was considered a failure). Nine (23.7%) of the procedures achieved PVI by QMODE+ only while 28 (73.7%) achieved PVI by both QMODE and QMODE+ . Of the non-PV ablations in 16 participants, cavotricuspid isthmus lines were ablated by QMODE in 9 participants, QMODE+ in 1 participant, and by both modes combined in 1 participant; QMODE+ was used for roof, posterior, and septal linear ablations in 6 participants. Protocol mandated activated clotting time ≥ 325 s was maintained throughout the procedure. The median (quartile 1/quartile 3) contact force was 13.8 g (12.5/16.2). Over a mean follow-up of 88 ± 8 days, there were no device-related adverse events. The study catheter was systematically examined for char after withdrawal; no char was observed upon visual inspection. One serious adverse event (1/39 [2.6%]) was reported in the safety analysis set, a fever requiring admission, which was procedure related. Seven procedure-related, nonserious adverse events were reported in 6 participants, including 1 pericardial effusion and 1 hematoma not requiring medical intervention.

Given the contrasting observation of catheter tip char versus prior reports with QMODE+ ablation [5, 6], an ad hoc analysis was performed to assess the relationship between baseline impedance and current change. Of 3352 analyzed lesions, 71.2% and 28.8% were delivered with QMODE+ and QMODE, respectively. Figure 1A shows the baseline impedance distribution across all lesions. Mean baseline impedance was 118.5 ± 5.1 Ω in 42.6% of QMODE+ lesions. Maximum current inversely correlated with baseline impedance for QMODE + ablations (Fig. 1B), ranging from 0.95 to 0.87 A when the baseline impedance was in the optimal range of 110 to 130 Ω and > 0.95 A when baseline impedance was < 110 Ω. Figure 1C shows an example of the current profile during 90 W, 50 W, and 30 W ablation delivery in a single participant. The increase in current in response to 90 W ablation was within 1 s, with fluctuation based on target catheter tip temperature and power, suggesting fast ramp up. Among ablatons performed with QMODE+ , QMODE/50 W, and QMODE/30 W, the mean maximum current observed was 0.95 ± 0.05 A, 0.7 ± 0.04 A, and 0.62 ± 0.03 A, respectively.

**Fig. 1:**
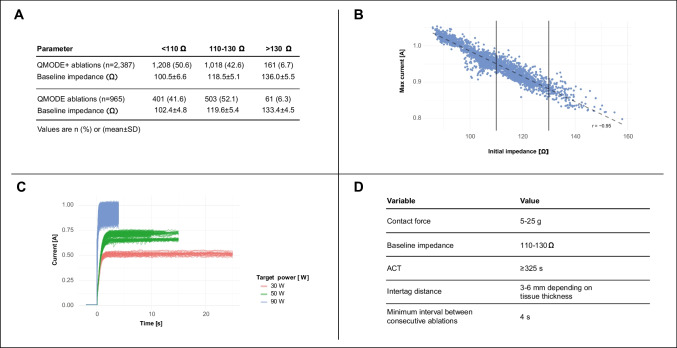
**A** Baseline impedance distribution for QMODE+ and QMODE lesions. **B** Scatter plot representing inverse linear association between maximum current (y-axis) and baseline impedance (x-axis) for the 2387 lesions QMODE+ delivered during the study. The data are divided into 3 groups: baseline impedance < 110 Ω, optimal baseline impedance between 110 and 130 Ω, and baseline impedance > 130 Ω. **C** Current profile in a single study participant during QMODE+ (90W), QMODE (50W and 30W) ablations. **D** Recommended optimal ablation and workflow settings based on this experience. ACT, activated clotting time

Ablation lesions are formed when RF energy is delivered against resistive myocardial tissue. The resulting current is dependent on the power used and circuit impedance. Overheating due to excess current delivery can compromise safety [7]. Lesion size of overheated tissue has been reported to be significantly larger with lower circuit impedance [7]. Indeed, Mueller et al. observed greater catheter tip coagulum in patients with lower baseline impedance, albeit nonstatistically significant [6]. Additionally, this finding was resolved after repositioning the dispersive electrode to increase baseline impedance from 90 to 110 Ω [6].

Although our observation of no catheter tip char contrasts with previous reports, it clinically validates the inverse association between baseline impedance and current flow with QMODE+ ablation [5, 6]. Interestingly, although no char was observed, 51% of QMODE+ lesions had a baseline impedance < 110 Ω, suggesting that additional factors may contribute to tissue overheating and char formation, such as contact force, catheter irrigation, intertag spacing, time interval between adjacent lesions, and anticoagulation.

To our knowledge, this is the first clinical report of the correlation between baseline impedance and current rise with QMODE+ ablation. Figure 1D lists the ablation settings observed in this clinical study for factors that may contribute to safety and particularly tip char minimization.

The limitations inherent in our study design include the small sample size, single arm, nonrandomized design, and lack of longer-term follow-up to assess ablation effectiveness.

In conclusion, the QDM catheter with the new nGEN generator demonstrated the acute safety and effectiveness of temperature-controlled RF ablation with no char observed. Careful consideration of multiple ablation settings may help minimize the risk of char and should be further investigated in future studies.

## Data Availability

Johnson & Johnson MedTech Companies have an agreement with the Yale Open Data Access (YODA) Project to serve as the independent review panel for the evaluation of requests for clinical study reports and patient-level data from investigators and physicians for scientific research that will advance medical knowledge and public health. Requests for access to the study data can be submitted through the YODA Project site at http://yoda.yale.edu.
